# TRPV4 differentially controls inflammatory cytokine networks during static and dynamic compression of the intervertebral disc

**DOI:** 10.1002/jsp2.1282

**Published:** 2023-10-27

**Authors:** Garrett W. D. Easson, Alireza Savadipour, Christian Gonzalez, Farshid Guilak, Simon Y. Tang

**Affiliations:** ^1^ Department of Orthopaedic Surgery Washington University in St. Louis St. Louis Missouri USA; ^2^ Department of Mechanical Engineering and Materials Science Washington University in St. Louis St. Louis Missouri USA; ^3^ Shriners Hospitals for Children—St. Louis St. Louis Missouri USA; ^4^ Department of Biomedical Engineering Washington University in St. Louis St. Louis Missouri USA

**Keywords:** degeneration, dynamic compression, inflammatory cytokine networks, intervertebral disc, static compression, TRPV4

## Abstract

**Background:**

The ion channel transient receptor potential vanilloid 4 (TRPV4) critically transduces mechanical forces in the IVD, and its inhibition can prevent IVD degeneration due to static overloading. However, it remains unknown whether different modes of loading signals through TRPV4 to regulate the expression of inflammatory cytokines. We hypothesized that TRPV4 signaling is essential during static and dynamic loading to mediate homeostasis and mechanotransduction.

**Methods:**

Mouse functional spine units were isolated and either cyclically compressed for 5 days (1 Hz, 1 h, 10% strain) or statically compressed (24 h, 0.2 MPa). Conditioned media were monitored at 6 h, 24 h, 2 days, and 5 days, with and without TRPV4 inhibition. Effects of TRPV4 activation was also evaluated without loading. The media was analyzed for a panel of 44 cytokines using a microbead array and then a correlative network was constructed to explore the regulatory relationships during loading and TRPV4 inhibition. After the loading regimen, the IVDs were evaluated histologically for degeneration.

**Results:**

Activation of TRPV4 led to an increase interleukin‐6 (IL‐6) family of cytokines (IL‐6, IL‐11, IL‐16, and leukemia inhibitory factor [LIF]) and decreased the T‐cell (CCL3, CCL4, CCL17, CCL20, CCL22, and CXCL10) and monocyte (CCL2 and CCL12) recruiting chemokines by the IVD. Dynamic and static loading each provoked unique chemokine correlation networks. The inhibition of TRPV4 during dynamic loading dysregulated the relationship between LIF and other cytokines, while the inhibition of TRPV4 during static loading disrupted the connectivity of IL‐16 and VEGFA.

**Conclusions:**

We demonstrated that TRPV4 critically mediates the cytokine production following dynamic and static loading. The activation of TRPV4 upregulated a diverse set of cytokines that may suppress the chemotaxis of T‐cells and monocytes, implicating the role of TRPV4 in maintaining the immune privilege of healthy IVD.

## Graphical abstract

TRPV4 critically and differentially mediates the cytokine production following dynamic and static loading. The activation of TRPV4 upregulated a diverse set of cytokines that may suppress the chemotaxis of T‐cells and monocytes, implicating the role of TRPV4 in maintaining the immune privilege of healthy IVD.

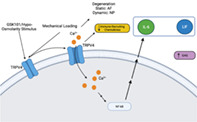

## 1 INTRODUCTION

Low back pain (LBP) is one of the leading causes of disability in the world and has poor long‐term treatment outcomes.^1^ A significant proportion of LBP is attributed to the degeneration of the intervertebral disc (IVD), the primary soft‐tissue component of the spine.^2^ Factors including aging, injury, and mechanical overloading, can cause IVD degeneration, which is often characterized by matrix degradation, nerve and blood vessel infiltration, and chronic inflammation.^3^, ^4^, ^5^, ^6^, ^7^, ^8^ Chronic inflammation can cause structural and signaling changes to the dorsal root ganglion and increase the presentation of pain.^9^

Cells of the IVD are responsive to mechanical loads, and mechanical loading is necessary for maintaining the homeostasis of the IVD.^10^, ^11^, ^12^, ^13^, ^14^ Physiologic loading conditions can promote glycosaminoglycan synthesis and promote cell viability,^14^, ^15^, ^16^ yet overloading or aberrant sustained loading can cause a cell‐mediated cascade that eventually leads to degeneration.^17^, ^18^ This cascade is often marked by the overexpression of matrix metalloproteinases, aggrecanases, and inflammatory cytokines. The transient receptor potential vanilloid 4 (TRPV4) ion channel has been shown to play a key role in transducing mechanical signals in cartilage and in IVD cells.^19^, ^20^ The inhibition of TRPV4 diminishes the expression of tumor necrosis factor‐α (TNF‐α) during hypo‐osmolarity,^21^ and the ablation of TRVP4 in annulus fibrosus (AF) cells reduces interleukin‐6 (IL‐6) and IL‐8 production during hyperphysiological stretching.^20^, ^21^, ^22^ At the tissue level, antagonizing TRPV4 during sustained loading protects against degeneration and blunts IL‐6 and VEGFA production.^23^

Activities of daily living, such as walking, running, and jumping, result in dynamic loading on the skeleton. These dynamic loading scenarios can be mimicked through cyclic loading at the tissue level to interrogate the role of TRPV4 during physiological loads. Though cellular‐level studies provided important insights on adaptations of specific IVD cells,^20^, ^24^, ^25^ the extracellular matrix (ECM) is critical for understanding whether physiologically relevant loads can invoke cellular responses that subsequently affect the degeneration and ECM organization. Therefore, in this study, we investigated the TRPV4‐mediated responses of the IVD during dynamic loading. We contrasted the cytokine profiles with static loading and the activation of TRPV4 in the IVD. Our results suggest that the inhibition of TRPV4 during dynamic loading partially protects against IVD degeneration. Correlation network analyses of the cytokines revealed that the IVD responds differently to dynamic and static loading, and the inhibition of TRPV4 dysregulates these relationships. Taken together, this work demonstrates that TRPV4 has disparate and crucial control in how the IVD produces immunomodulatory signaling proteins across different loading modes commonly experienced by the IVD.

## 2 METHODS

### 2.1 Animals

All procedures involving animals were carried out in accordance with the guidelines set by the Institutional Animal Care and Use Committee of Washington University in St. Louis. In this study, equal numbers of male and female 16‐week‐old mice were pooled into each group, which carried the nuclear factor kappa B (NF‐κB)‐luciferase reporter transgene (FVB.Cg‐Tg(HIV‐EGFP,luc)8Tb/J; JAX). Before the experiments, the mice were euthanized with CO_2_ for a duration of 5 min, and the incision sites were disinfected using 70% ethanol. The entire lumbar spine was then extracted and divided into three functional spine units (FSUs): L1/L2, L3/L4, and L5/L6. The spinal cord and extraneous tissues were removed from the FSUs. The FSUs were immediately placed in culture media in 24‐well plates and preconditioned for 7 days before the administration of the experimental conditions.^26^ The culture media consisted of a mixture of Dulbecco's modified Eagle's medium and nutrient mixture F‐12 in a 1:1 ratio (DMEM:F12), supplemented with 20% fetal bovine serum and 1% penicillin–streptomycin. The FSUs were cultured under controlled conditions of 37°C, 5% CO_2_, and 100% humidity. As TRPV4 is an osmotically sensitive channel, all culture media was intentionally maintained at 400 mOsm throughout the study. A total of 54 FSUs were used for these experiments. A schematic for the data collection strategies for the longitudinal and cross‐sectional measurements is shown in Figure S1.

### 2.2 Longitudinal dynamic and static compression in culture

A closed‐loop displacement‐controlled bioreactor was utilized to apply dynamic compression to FSUs in culture.^19^, ^27^ Deformation was applied to the FSUs by individual polyacetal pistons that are connected to a stage that is controlled by a stepper motor and custom LabView software. A uniform displacement of 35 μm, approximately 10% axial strain of the IVD height following preconditioning, was applied to the FSUs to load the IVD in a sinusoidal regimen at 2 Hz for 1 h per day for 5 consecutive days.^28^ We added either dimethyl sulfoxide (DMSO) (vehicle, *n* = 8) or 10 μM GSK205 (TRPV4 antagonist, *n* = 8) to the medium of compressed IVDs throughout the entire loading process. The loaded samples were compared to a free‐swelling control with DMSO (*n* = 8).^29^

To compare to dynamic compression, samples were subjected to static compression as previously described.^23^ Static loading of the FSUs were conducted using a platen‐spring apparatus that applied approximately 0.2 MPa for 24 h. During the loading period, samples were either treated with DMSO (*n* = 6) or TRPV4 antagonist GSK205 (10 μM, *n* = 6). Following the 24‐h loading period, the device was removed, and samples were cultured for two additional days. Media was then collected for cytokine analysis. Previous work has shown that 10.3 ± 3.5% axial strain corresponds approximately to 0.35 ± 0.22 MPa peak stress.^30^ Thus, the two loading modes in this study are intended to be approximately equal in magnitude.

### 2.3 NF‐κB imaging

NF‐κB signaling was measured in the IVD cells 6 h post‐loading to confirm that dynamic compression induces a biological response, a known peak for NF‐κB signaling following TRPV4 activation.^23^, ^31^ Luciferin (30 mg/mL) was added to the culture to bind to the luciferase transgene. Bioluminescence was measured for 30 min following luciferin addition using an IVIS Imaging System (Xenogen Corp.) at a 10‐s exposure time.

### 2.4 Cytokine analyses

The concentration of IL‐6 in the media on Days 0, 2, and 5 was measured by ELISA (Mouse IL‐6 ELISA kit; Thermo Fisher Scientific). Cytokines following two bouts of dynamic compression were measured by Luminex multiplex bead assays. We chose Day 2 to analyze as this was the peak response in IL‐6 in the 5‐day time course. The media were analyzed using the Luminex™ 200 system (Luminex) by Eve Technologies Corp. A total of 44 markers were simultaneously measured in the samples using Eve Technologies' Mouse Cytokine 44‐Plex Discovery Assay®, which consists of two separate kits; one 32‐plex and one 12‐plex (MilliporeSigma). The assay was conducted in accordance with the manufacturer's protocol. The 32‐plex consisted of Eotaxin (CCL11), granulocyte colony‐stimulating factor (GCSF), granulocyte‐macrophage colony‐stimulating factor (GMCSF), IFN‐γ, IL‐1α, IL‐1β, IL‐2, IL‐3, IL‐4, IL‐5, IL‐6, IL‐7, IL‐9, IL‐10, IL‐12 (p40), IL‐12 (p70), IL‐13, IL‐15, IL‐17, IP‐10 (CXCL10), KC (CXCL1), leukemia inhibitory factor (LIF), LIX (CXCL5), MCP‐1 (CCL2), MCSF, MIG (CXCL9), MIP‐1α (CCL3), MIP‐1β (CCL4), MIP‐2 (CXCL2), RANTES (CCL5), TNF‐α, and VEGFA. The 13‐plex consisted of 6Ckine/Exodus2 (CCL21), Fractalkine (CX3CL1), IFN‐β1, IL‐11, IL‐16, IL‐20, MCP‐5 (CCL12), MDC (CCL22), MIP‐3α (CCL20), MIP‐3β (CCL19), TARC (CCL17), and TIMP‐1. Assay sensitivities of these markers range from 0.3–30.6 pg/mL for the 45‐plex. Individual analyte sensitivity values are available in the MilliporeSigma MILLIPLEX® MAP protocol. Chemokines are reported by their CCL and CXCL naming conventions based on the number and spacing of conserved cysteines.

### 2.5 Histology

All samples were formalin‐fixed and embedded in paraffin. Samples were sectioned into 10 μm thick sections and stained with Safranin O, followed by a Fast Green counterstain. To assess IVD degeneration, a standardized scoring system was employed, and a single, blinded grader scored the IVDs on 3 nonconsecutive days.^32^

### 2.6 Cytokine network analysis

A correlative network was used to identify regulatory relationships between measured cytokines. The correlation networks were created using Pearson's correlations between each cytokine. Correlations between all cytokines were determined in RStudio, and cytokine networks were plotted in MATLAB 2018. Shown correlations for the network are either *r* > 0.9 (Dynamic and Static Compression) or *r* > 0.7 (TRPV4 activation). Correlations greater than 0.9 are considered “very strong” and correlations greater than 0.7 are considered “strong.”^33^ Cytokines below detectable thresholds were not included in the analysis. The influence of individual cytokines on their respective networks was also quantified via eigenvector centrality (C_λ_), wherein well‐connected and powerful nodes have higher numerical values. C_λ_ is bounded by a 0–1 scale, in which 0 represents no connections and 1 would be significant correlations with every other cytokine in the panel.

### 2.7 Activation of TRPV4 in IVDs

We examined the effects of TRPV4 activation on cytokine production. The FSUs were incubated with either DMSO (*n* = 6) or the TRPV4 agonist GSK1016790A (GSK101, 2 μM; *n* = 10) for 3 h each day for 7 consecutive days. Following the 3 h, IVDs were returned to fresh media. Media was collected on Days 3 and 7 and analyzed using multiplex cytokine assay.

### 2.8 Statistics

All statistical analysis was completed in either RStudio, MATLAB, or GraphPad Prism 9 software. We conducted comparisons across experimental factors using either Student's *t*‐test or a multi‐way analysis of variance. Post hoc comparisons between groups were conducted using Tukey's adjusted method. Results were considered statistically significant when the *p* < 0.05.

## 3 RESULTS

### 3.1 Dynamic compression invokes an acute inflammatory response

**FIGURE 1:**
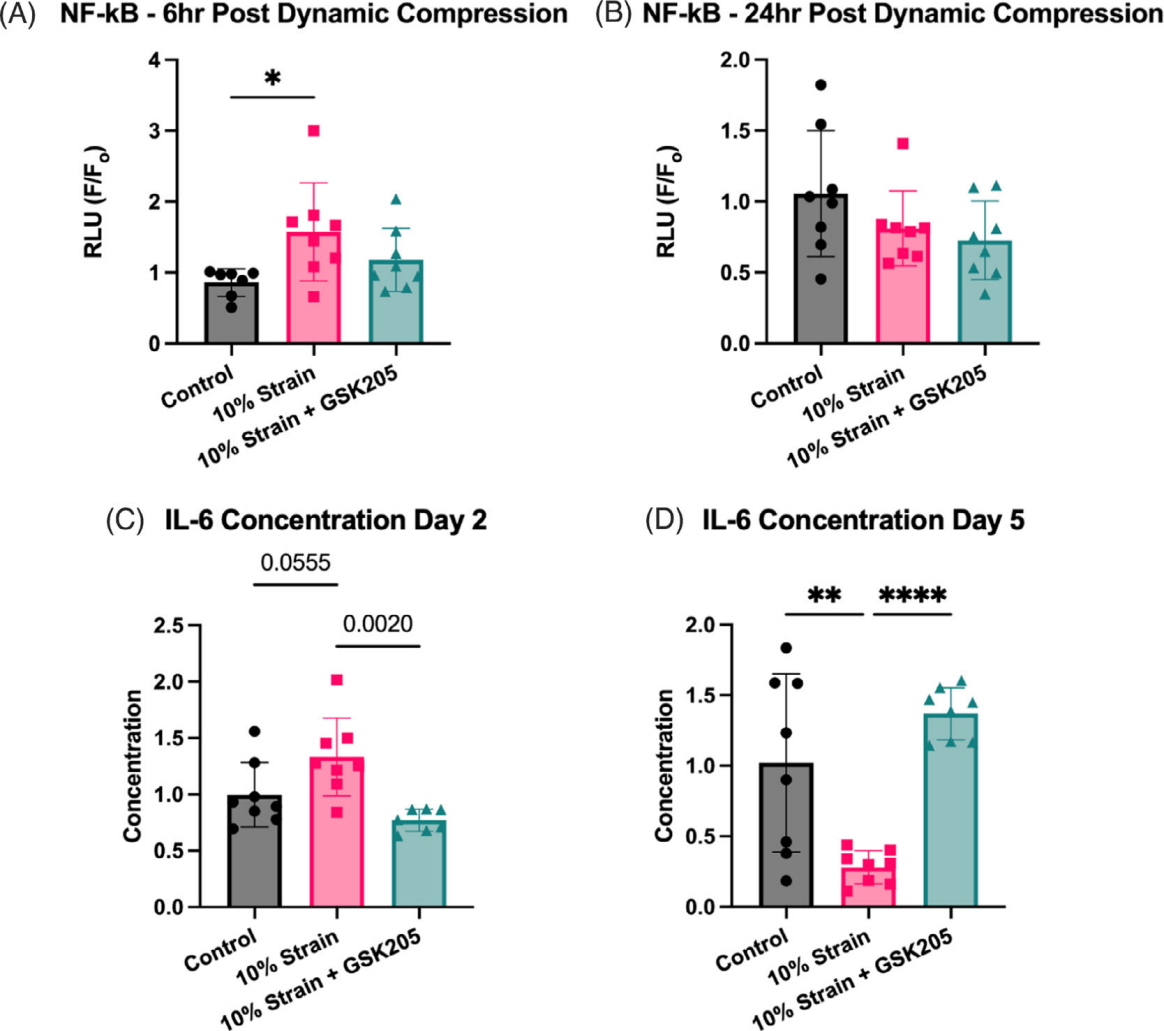
(A) Dynamic compression activated nuclear factor kappa B (NF‐κB) (*p* < 0.05), confirming that this a single bout of this loading magnitude and frequency evokes a biological response. (B) The NF‐κB signaling response subsided after 24 h. (C) Two days following the dynamic interleukin‐6 (IL‐6) further confirmed inflammation resulted from dynamic compression, which is absent with transient receptor potential vanilloid 4 (TRPV4) inhibition. (D) After 5 days of loading, IL‐6 concentration was significantly down (*p* < 0.05) and compressed intervertebral discs with TRPV4 inhibited remained unchanged. Data were statistically analyzed using one‐way analysis of variance with post hoc Tukey's HSD where *p < 0.05; **p < 0.005; *** *p* < 0.001; **** *p* < 0.0001.

Dynamic compression enhanced NF‐κB signaling 6 h post‐loading (*p* < 0.05), confirming that the loading evoked a biological response in the FSU (Figure 1A). Twenty‐four hours post‐loading, the levels of NF‐κB signaling were indistinguishable from unloaded levels (Figure 1B). Inhibition of TRPV4 by GSK205 did not significantly reduce the response to loading. After two bouts of loading, IL‐6 concentration in the media increased nonsignificantly (*p* = 0.056), and TRPV4‐inhibition blunted this response (Figure 1C). Following the entire 5‐day loading regimen, there was a significant decrease in IL‐6 concentration in the dynamically compressed group (Figure 1D). Metabolic activity, as measured by Alamar Blue, was unchanged (Figure S2).

### 3.2 IL‐1 and neutrophil‐recruiting chemokines are elevated following loading

**FIGURE 2:**
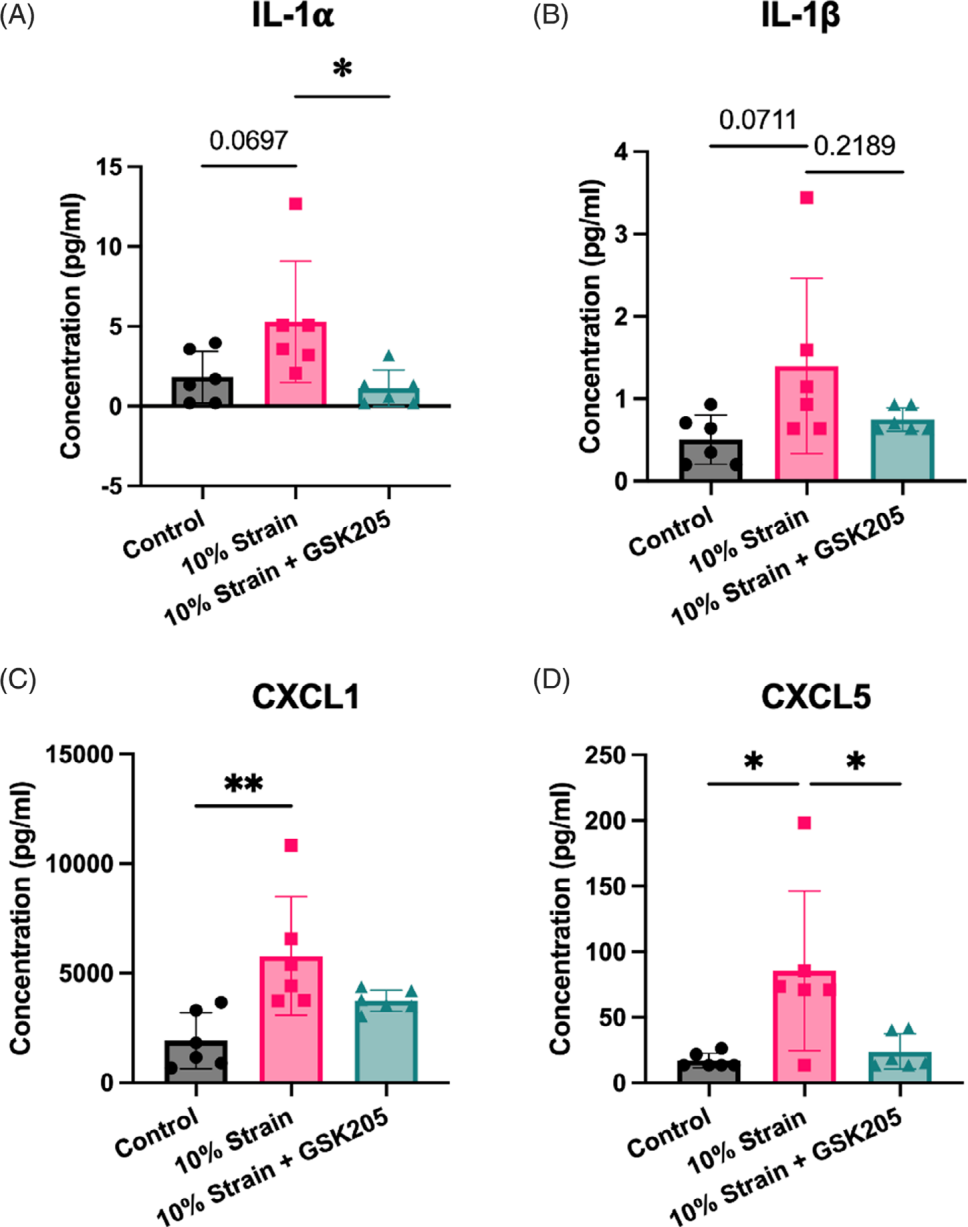
(A) Interleukin‐1α (IL‐1α) and (B) IL‐1β trended toward significant increases (*p* = 0.0697; *p* = 0.0711) with dynamic compression, and the transient receptor potential vanilloid 4 (TRPV4) inhibition prevented the IL‐1α production mediated by dynamic compression. (C, D) Neutrophil‐recruiting chemokines CXCL1 and CXCL5 were elevated after 2 days of loading, and only CXCL5 was blunted by TRPV4 inhibition. Data were statistically analyzed using one‐way analysis of variance with post hoc Tukey's HSD where *p < 0.05; **p < 0.005; and so forth.

Cytokine array analysis after 2 days of dynamic loading revealed nonsignificant increases of IL‐1α and IL‐1β (*p* = 0.0697; *p* = 0.0711; Figure 2A,B). TRPV4 inhibition during load reduced the production of IL‐1α but did not have a significant effect on IL‐1β levels. The neutrophil‐recruiting chemokines, CXCL1 and CXCL5, were both significantly increased following dynamic compression, and TRPV4 inhibition protected against the increase in CXCL5 (Figure 2C,D).

### 3.3 Dynamic loading causes degenerative changes localized to the nucleus pulposus

**FIGURE 3:**
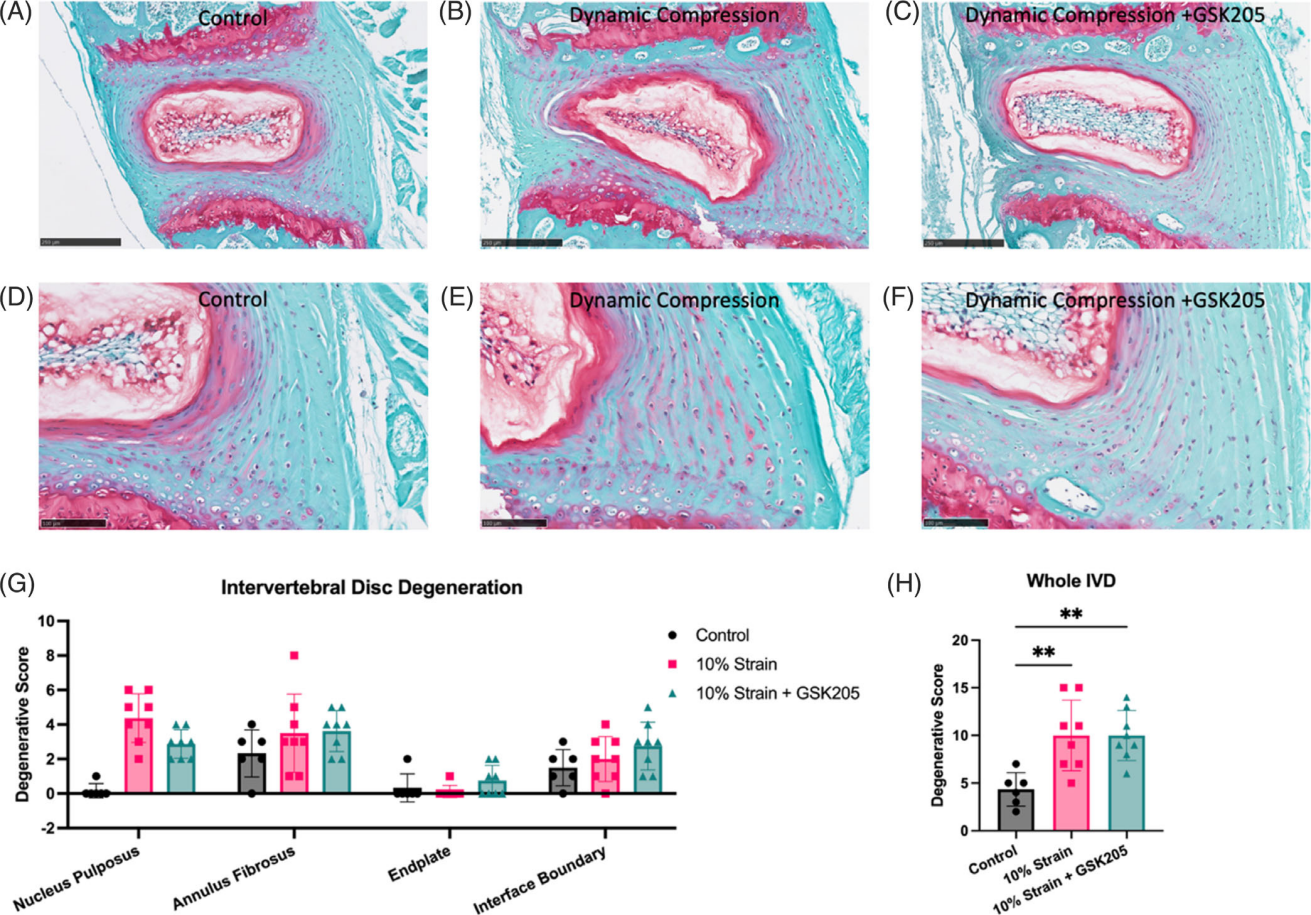
Intervertebral discs (IVDs) subjected to 5 days of dynamic compression (1 h/day, 1Hz, 10% compressive strain) exhibited bulging in the nucleus pulposus (NP), altered NP shape, mild fibrosis and NP cell reduction when compared to control IVDs (A, D and B, E). Inhibiting transient receptor potential vanilloid 4 (TRPV4) during compression (C, F) prevented these degenerative changes. (G) The degenerative changes in the NP were most apparent, as the compressed IVDs were significantly more degenerated than controls (*p* < 0.05). Other compartments were unaffected by the dynamic loading regimen. (H) When summing all compartments together, inhibiting TRPV4 did not have an effect on the overall degenerative changes caused by dynamic compression. Scale bar = 100 μm. Data were statistically analyzed using one‐way analysis of variance with post hoc Tukey's HSD where *p < 0.05; **p < 0.005; and so forth.

Histologic evaluation of the IVDs following 5‐days of dynamic compression revealed degenerative changes occurring in the nucleus pulposus (NP), including changes in the NP shape, decreased cell count, and mild fibrosis (Figure 3A–F). Inhibiting TRPV4 reduced degeneration in the IVDs. The degenerative changes were localized in the NP but not in the other IVD compartments (Figure 3G). When summed together, the degenerative scoring resulted in a significant increase in the compressed IVD that was not altered with TRPV4 inhibition (Figure 3H).

### 3.4 Cytokine network differs between static and dynamic compression

**FIGURE 4:**
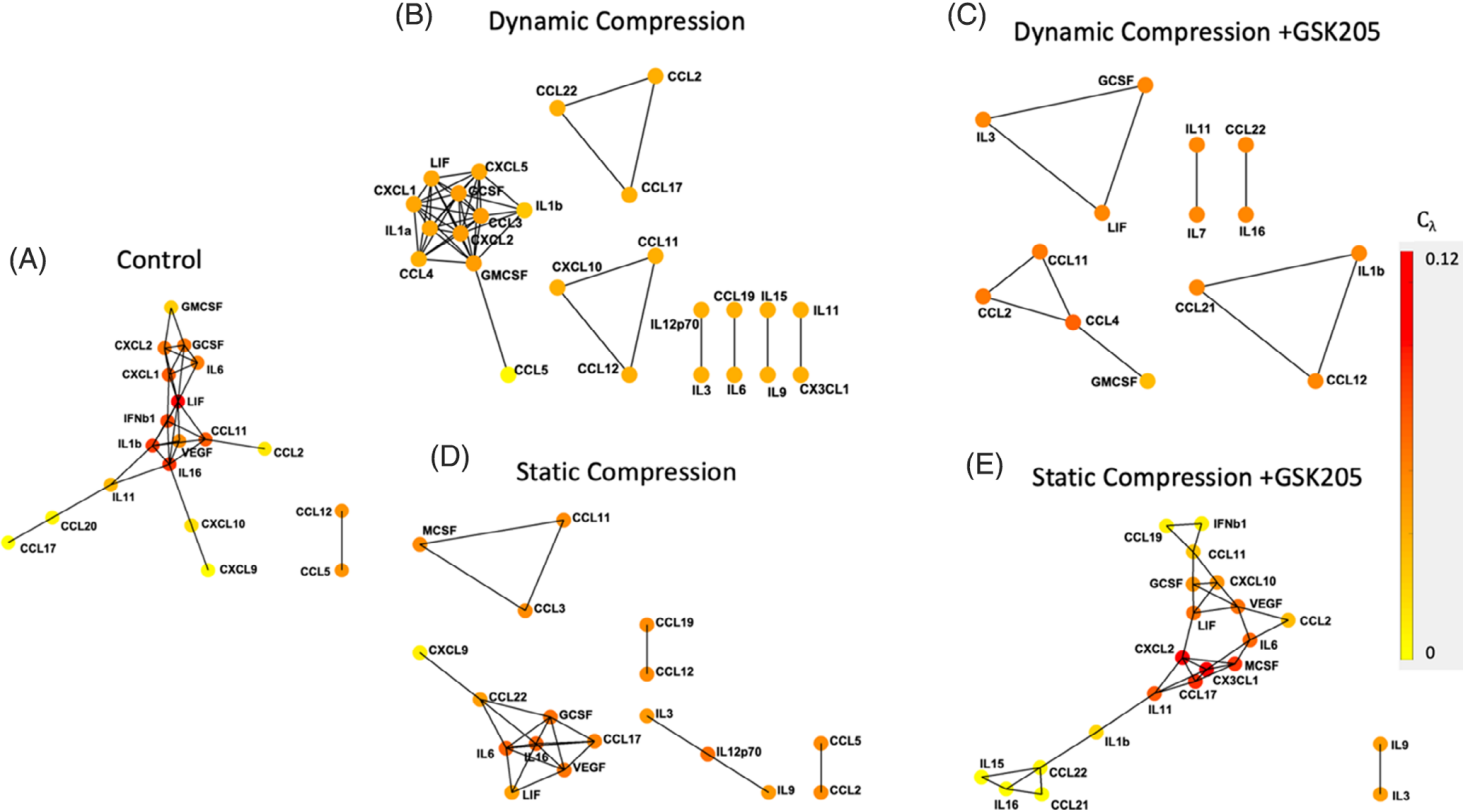
Cytokine network analysis between control, statically, and dynamically compressed samples, with and without transient receptor potential vanilloid 4, showed notable changes including those cytokines that correlate with leukemia inhibitory factor and the positive correlation between interleukin‐16 and VEGF in static loading. All correlations coefficients (*r*) are > 0.9.

Cytokine networks were constructed for control (*n* = 6), dynamic compression (*n* = 6), dynamic compression + GSK205 (*n* = 6), static compression (*n* = 6), and static compression + GSK205 (*n* = 7) (Figure 4). In the control network, the IL‐6 family of cytokines, including IL‐6, LIF, IL‐11, and IL‐1β, are highly correlated (Figure 4A). Members of this pathway are also intercorrelated with CXCL1 and CXCL2, canonical neutrophil‐recruiting chemokines. LIF and IL‐16 are inversely correlated with VEGF in these control conditions. The cytokine array results were in excellent agreement with the ELISA results (Figure S2).

Dynamic compression preserved the correlations between LIF and CXCL1, CXCL2, GCSF, and GMCSF (Figure 4B). Dynamic compression introduced novel connections between LIF with IL‐1α and CXCL5, both of which were significantly increased during dynamic loading. Dynamic compression also disrupted the canonical connectivity between LIF and IL‐6. The inhibition of TRPV4 in dynamic compression maintained the correlations between LIF:GCSF and CCL4:GMCSF (Figure 4C). TRPV4 inhibition also disrupted the LIF connections with IL‐1α and CXCL5 that manifested following compression, as well as the LIF connections with CXCL1 and CXCL2.

The correlative cytokine networks in both the control and the static loading groups exhibited the three‐way connection between LIF, IL‐16, and VEGF (Figure 4A,D). Notably, though, the inverse relationship that both LIF and IL‐16 had with VEGF in the control group was reversed following static loading and became significantly positive. With TRPV4 inhibition, the VEGF correlations with LIF and IL‐6 were maintained and remained positive, though LIF and IL‐6 were no longer correlated with each other (Figure 4E). The IL‐16 and CCL22 connection was also preserved.

The static compression and dynamic compression cytokine networks varied dramatically. None of the correlations between LIF nor GCSF overlapped between the two loading groups. The correlation of IL‐3:IL‐12p70 was the only similarity between the two groups. All cytokine results are in Tables S1 and S2. All correlations in all groups are provided in Tables S3–S9.

### 3.5 IL‐6 family of cytokines secreted from TRPV4 activation

**FIGURE 5:**
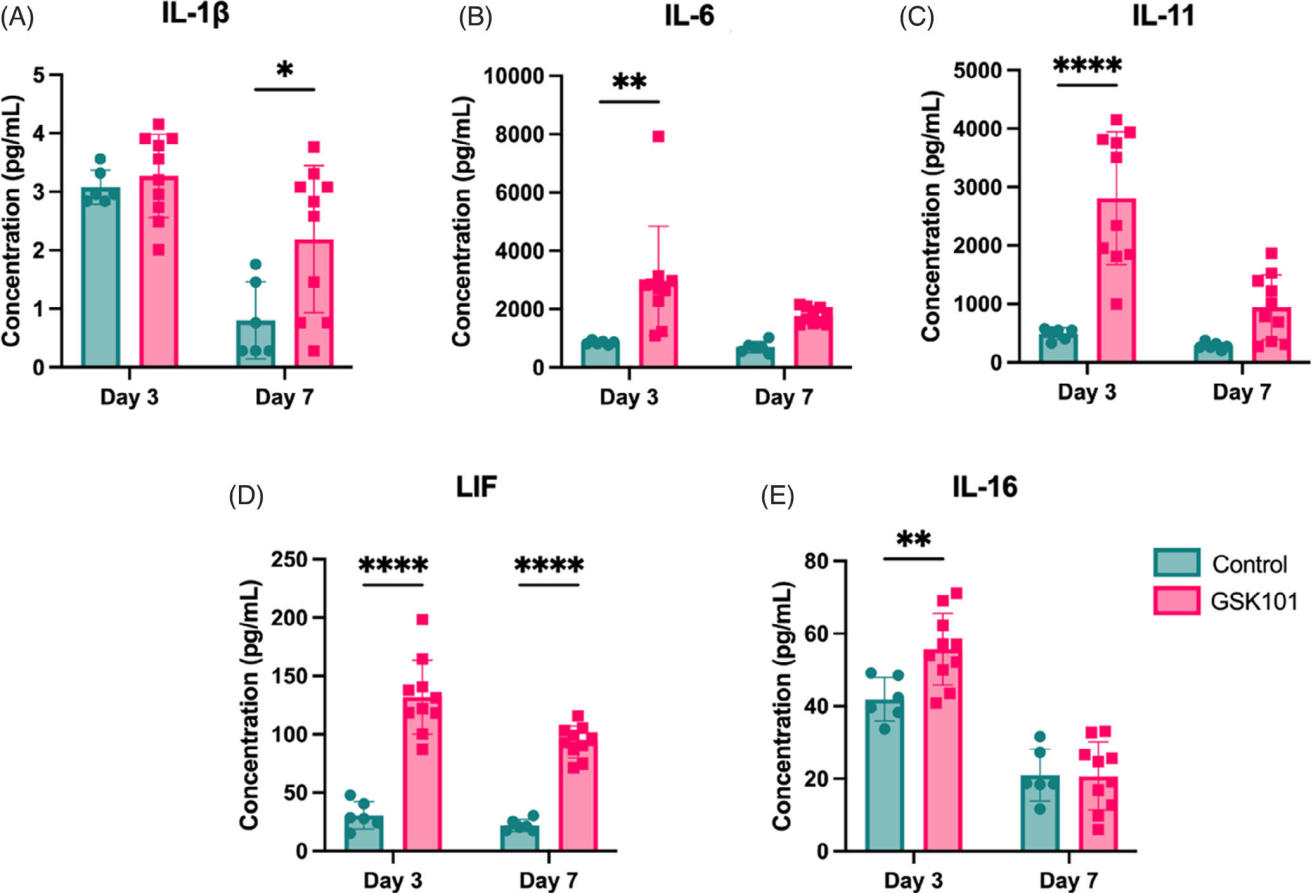
Repeated transient receptor potential vanilloid 4 activation by GSK101 administration increased interleukin‐1β (IL‐1β) production (A) at Day 7 but not at Day 3. IL‐6 (B), IL‐11 (C), and IL‐16 (E) all had higher concentration at the acute Day 3 timepoint, but were not significantly increased at Day 7. Leukemia inhibitory factor (D) was the only cytokine elevated at both days. Data were statistically analyzed using two‐way analysis of variance with post hoc Tukey's HSD where *p < 0.05; **p < 0.005; and so forth.

Repeated TRPV4 activation increased cytokine production of IL‐6, IL‐11, LIF, and IL‐16 after 3 days (Figure 5B–E). Following 7 days of activation, IL‐1β and LIF were significantly up (Figure 5A,D).

### 3.6 TPRV4 activation diminishes chemokine production

**FIGURE 6:**
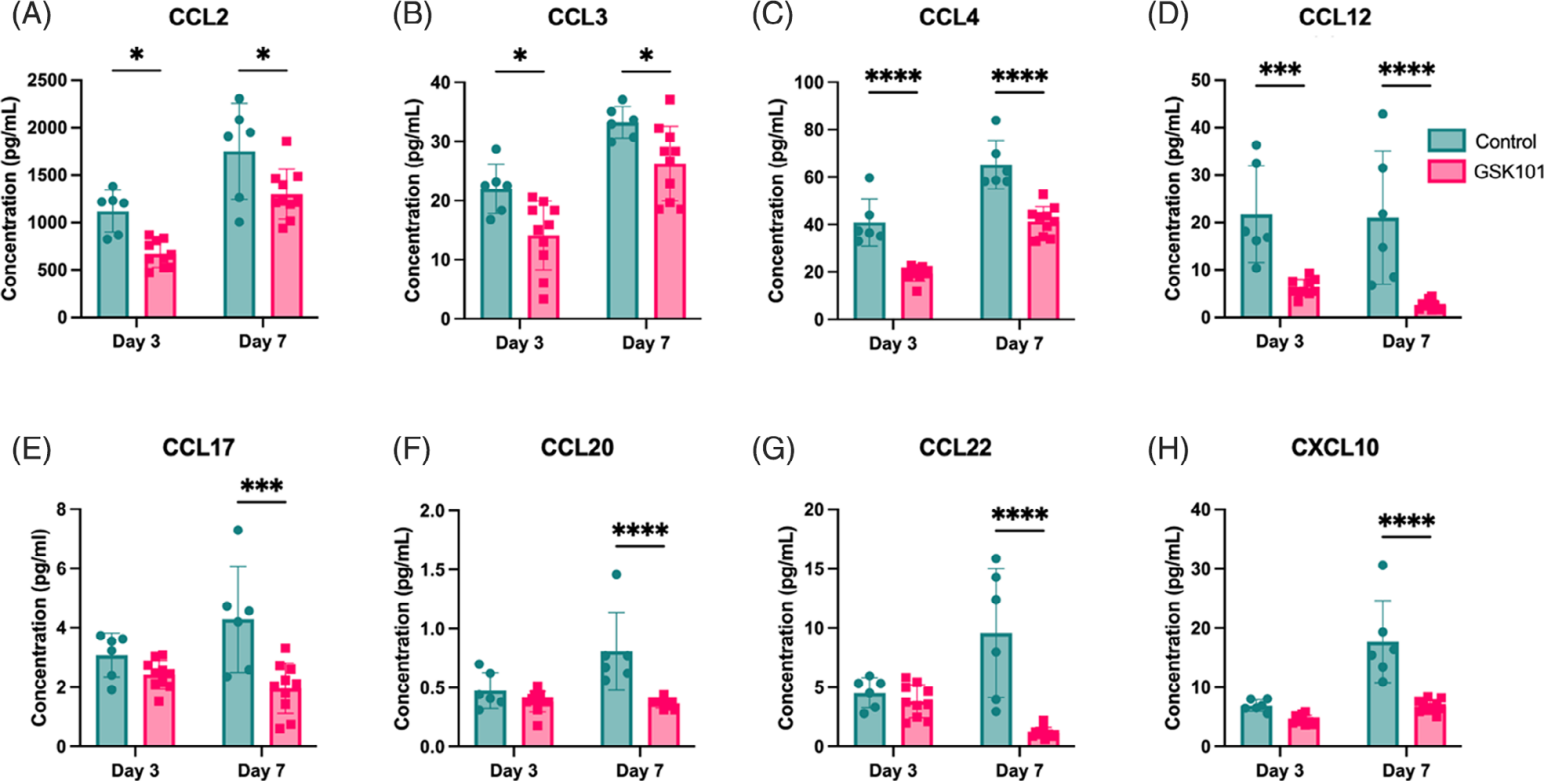
The monocyte‐recruiting chemokines CCL2 (A) and CCL12 (D) were both significantly decreased at Days 3 and 7 following repeated transient receptor potential vanilloid 4 (TRPV4) activation. Chemokines CCL3 (B) and CCL4 (C), which regulate T‐cell chemotaxis, were also decreased for the duration of the TRPV4 activation. Additional T‐cell recruiting chemokines CCL17 (E), CCL20 (F), CCL22 (G), and CXCL10 (H) were decreased at Day 7, indicating TRPV4 activation may prevent T‐cell infiltration. Data were statistically analyzed using two‐way analysis of variance with post hoc Tukey's HSD where *p < 0.05; **p < 0.005; and so forth.

Monocyte‐recruiting chemokines CCL2 and CCL12 were significantly decreased on both Day 3 and Day 7 of GSK101 administration (Figure 6A,D). CCL3 and CCL4, T‐cell recruiting chemokines, were also both decreased on Days 3 and 7 (Figure 6B,C). Following 7 days of TRPV4 activation, the T‐cell recruiters CCL17, CCL20, CCL22, and CXCL10 were suppressed (Figure 6E–H).

### 3.7 TRPV4‐activated cytokine network changes over time

**FIGURE 7:**
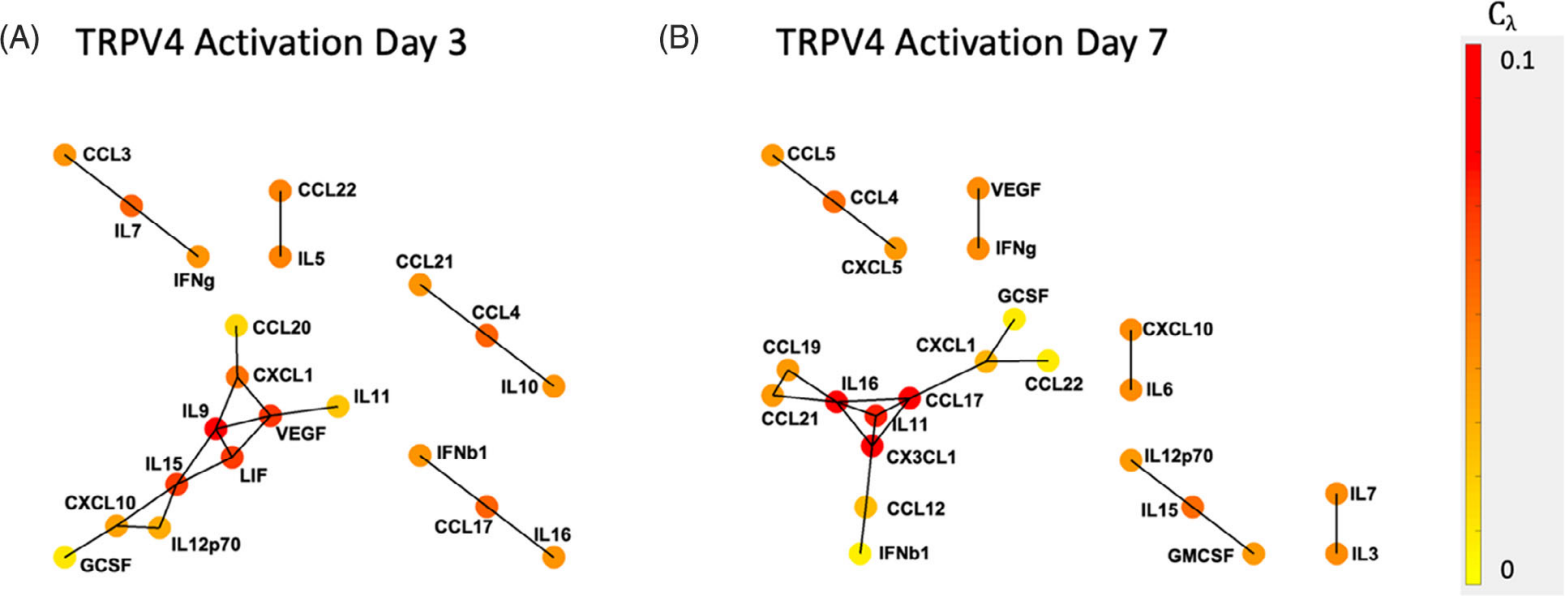
Repeated activation of transient receptor potential vanilloid 4 (TRPV4) results in temporally distinct cytokine correlation networks. The conserved correlations are limited to IL‐16:CCL17 and IL‐15:IL‐12p70 indicate that repeated TRPV4 activation dramatically alters cytokine production and has both an acute and chronic inflammatory phase. All correlation coefficients (*r*) are > 0.9.

Additional cytokine networks were created for IVDs for both Days 3 and 7 of repeated TRPV4 activation (Figure 7). Between these timepoints, IL‐16:CCL17 and IL‐15:IL‐12p70 correlations were conserved. At Day 3, cytokines IL‐11 and LIF correlated with VEGF. At the Day 7 timepoint, IL‐6 and CXCL10 are inversely related, and IL‐16 and IL‐11 are positively correlated.

## 4 DISCUSSION

The IVD responds to dynamic and static compression differently by producing chemokine profiles that are distinct and uniquely interrelated. TRPV4 appears to be required to transduce these load‐mediated responses. Dynamic compression invoked an acute NF‐κB signaling response at 6 h post‐loading that was resolved by 24 h. We have previously shown that the activation of TRPV4 activates intracellular calcium signaling and the subsequent intracellular NF‐κB binding activity.^19^, ^23^ Other studies have shown cascade invoked by calcium signaling.^34^, ^35^ While we did not measure all steps of this cascade, we have measured the crucial cellular intermediatories of NF‐κB binding. Downstream of NF‐κB signaling, IL‐6 cytokine secretion was nonsignificantly increased on Day 2 of dynamic compression and then decreased following 5 days of dynamic compression, suggesting that prolonged dynamic loading may be favorable for the control of IL‐6. At this peak, IL‐6 secretion, IL‐1α, and IL‐1β, as well as neutrophil‐recruiting chemokines CXCL1 and CXCL5, were also elevated. The cytokine correlation networks from the cytokine panel for dynamic and static compression exhibited some similarities between the two loading modes. LIF is a primary pathway that is preserved across loading modes and TRPV4 inhibition, and it emerged with new connective nodes due to dynamic loading. Static compression with and without TRPV4 inhibited the correlative changes to the IL‐6/VEGF/LIF networks. Repeated TRPV4 activation resulted in significant increases in the IL‐6 family cytokines as well as significant decreases in immune cell‐recruiting chemokines, suggesting that the frequency of TRPV4 activation may drive differential signaling profiles in the IVD.

We investigated the cytokine response to dynamic loading after 2 days of loading at peak IL‐6 concentration to elucidate the characteristics of the inflammatory response of the IVD. The culmination of dynamic loading resulted in mild degenerative changes to the NP, including gross morphologic changes to NP shape, lower cell counts in the NP, and fibrosis in the NP compartment. Inhibition of TRPV4 mitigated the degenerative response in the NP. In contrast, our previous report showed that static loading caused degenerative changes in all compartments of the IVD.^23^ The inhibition of TRPV4 through GSK205 during dynamic compression did not prevent degenerative changes from occurring in the AF and end plates, as degeneration was not significantly different from the dynamic compression group.

Dynamic loading increased production of CXCL1 and CXCL5, and both chemokines bind with CXCR2 to initiate neutrophil recruitment.^36^ IVD cells have been shown to secrete chemokines that functionally recruit intact neutrophils under challenge.^37^ Neutrophils are a part of the innate immune response and mediate inflammation.^38^ Furthermore, both CXCL1 and CXCL5 were significantly correlated with CXCL2, LIF, CCL3, GCSF, and GMCSF. With TRPV4 inhibition, CXCL1, CXCL2, and CXCL5 no longer correlated with each other, suggesting that TRPV4 may be required to coordinate these responses. In our study, static compression altered the inverse correlations of LIF and IL‐16 with VEGF into a positive correlation (Tables S2–S8). LIF appears to have versatile roles as it reduces VEGF expression in some cells while promoting VEGF production in others.^39^, ^40^, ^41^, ^42^ Accordingly, LIF differentially regulates key cell activities such as proliferation, apoptosis, and differentiation dependent on cellular context.^41^ Similar to dynamic compression, TRPV4 inhibition during static loading disassociated LIF from chemokines despite being secreted at high levels. Taken together, the TRPV4‐dependent response of the IVD to produce immune cell recruiting chemokines due to loading may be driven by LIF.

The activation of TRPV4 increased IL‐6, IL‐11, and IL‐16 concentration at the early timepoint (3 days post activation). These cytokines play roles in acute inflammation, and the sustained presence of IL‐6 in the IVD is strongly linked to chronic inflammation and IVD degeneration.^43^, ^44^, ^45^ Acute inflammation is necessary for tissue healing, implicating the inflammatory response of TRPV4 activation to regenerative.^46^, ^47^ This finding further supports the hypothesis that TRPV4 activation can induce matrix synthesis, which has been shown in the chondrocytes and IVD cells.^19^, ^22^, ^23^, ^31^, ^48^

Similar to our prior study,^23^ the TRPV4 activation increased IL‐6 by nearly threefold, though not statistically significant (*p* = 0.12). On Day 7, IL‐1β remains elevated. As IL‐1β and IL‐6 are often activated by similar mechanisms, it is likely that TRPV4 is an upstream regulator of these cytokines. The sustained, elevated levels of these cytokines are strongly associated with chronic inflammatory and degenerative pathways in the IVD. LIF production was also sustained with TRPV4 activation. Phillips et al. observed decreased levels of expression in degenerated NP cells.^49^ Another study reported increases in LIF in mildly degenerated tissue.^50^ In the same study, LIF stimulated aggrecan and Col2a1 matrix synthesis and inhibited NP apoptosis. In contrast, LIF appears to inhibit proteoglycan synthesis in chondrocytes.^51^, ^52^

Despite increasing inflammatory cytokine release, TRPV4 activation dampened the release of immune cell‐recruiting chemokines, implicating TRPV4 as a mediator of IVD‐immune cell crosstalk. CCL2 and CCL12 have many roles in the immune system but are primarily known for monocyte chemotaxis.^53^, ^54^, ^55^ These, along with T‐cell recruiters CCL3 and CCL4, were down at both timepoints.^56^ CCL3 and CCL4 are expressed by NP cells and have elevated expression in degenerated discs.^57^ CCL17, CCL20, CCL22, and CXCL10 all contribute to T‐cell chemotaxis.^58^, ^59^, ^60^, ^61^ The reduction of these T‐cell recruiting chemokines on Day 7 indicates TRPV4 activation may be protective against the infiltration of delayed T‐cells into the IVD. It has been shown that at 2 weeks post‐injury in a rat model of IVD degeneration and LBP, immune cells, including T‐cells, are significantly increased.^62^ Based on our findings here, there is potential to mediate this immune cell infiltration using controlled TRPV4 activation.

**FIGURE 8:**
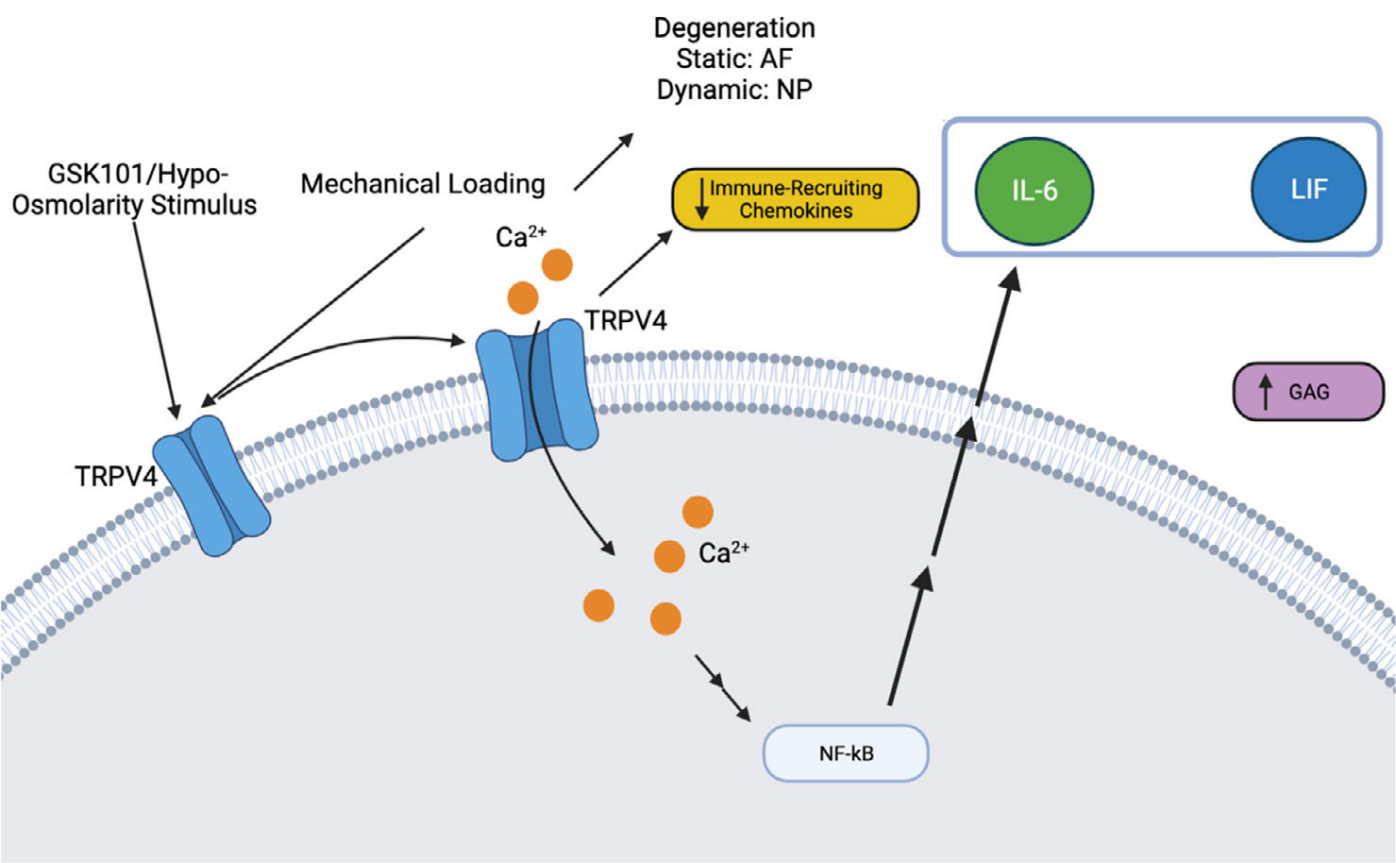
Overview of transient receptor potential vanilloid 4 (TRPV4)'s role in the intervertebral disc. Mechanical dynamic loading led to degenerative changes in the nucleus pulposus, while mechanical static loading led to degeneration in the annulus fibrosus. TRPV4 mediated these responses and differentially regulated the subsequent chemokine networks. Furthermore, TRPV4 activation promotes the IL‐6/leukemia inhibitory factor (LIF) pathway and decreases the secretion of immune cell‐recruiting chemokines.

The reduced chemokine production observed in this study further suggests that TRPV4 activation may have some protective capacities in the IVD. Taken with static and dynamic compression findings, the data suggest that the transient activation of TRPV4 induces an acute, proregenerative inflammatory response (Figure 8). The overproduction of inflammatory cytokines as mediated through the TRPV4 may be contributing to mechanically induced degenerative changes in the IVD.

## Supporting information


**DATA S1.** Supporting Information.
