# Supplementary material for: TRPV4 differentially controls inflammatory cytokine networks during static and dynamic compression of the intervertebral disc
Source: JOR Spine. 2023 Oct 27;6(4):e1282. doi: 10.1002/jsp2.1282 (PMC10751971; doi:10.1002/jsp2.1282)

**TRPV4 Differentially Controls Inflammatory Cytokine Networks During Static and Dynamic Compression of the Intervertebral Disc**

Garrett W. D. Easson^1,2^, Alireza Savadipour^1,2,3^, Christian Gonzalez^4^, Farshid Guilak^1,2,3,4^, Simon Y. Tang^1,2,4^

^1^Department of Orthopaedic Surgery, Washington University in St. Louis

^2^Department of Mechanical Engineering and Materials Science, Washington University in St. Louis

^3^Shriners Hospitals for Children – St. Louis

^4^Department of Biomedical Engineering, Washington University in St. Louis

**Keywords: TRPV4; Intervertebral Disc; Static Compression; Dynamic Compression; Inflammatory cytokine networks; Degeneration**

**Supplemental Materials
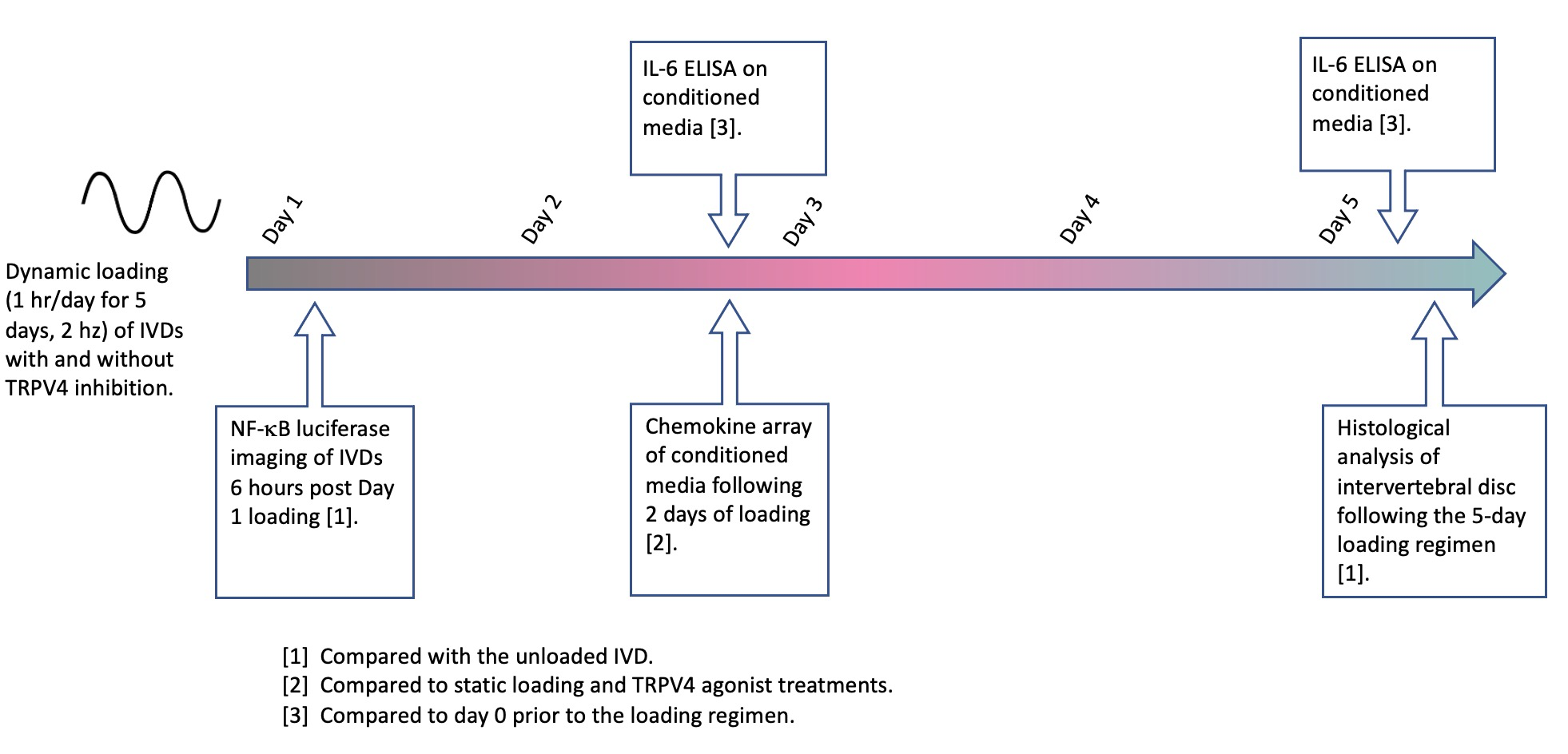
**

**Supplemental Figure 1:** A schematic for the data collection strategies for longitudinal and cross-sectional time points.


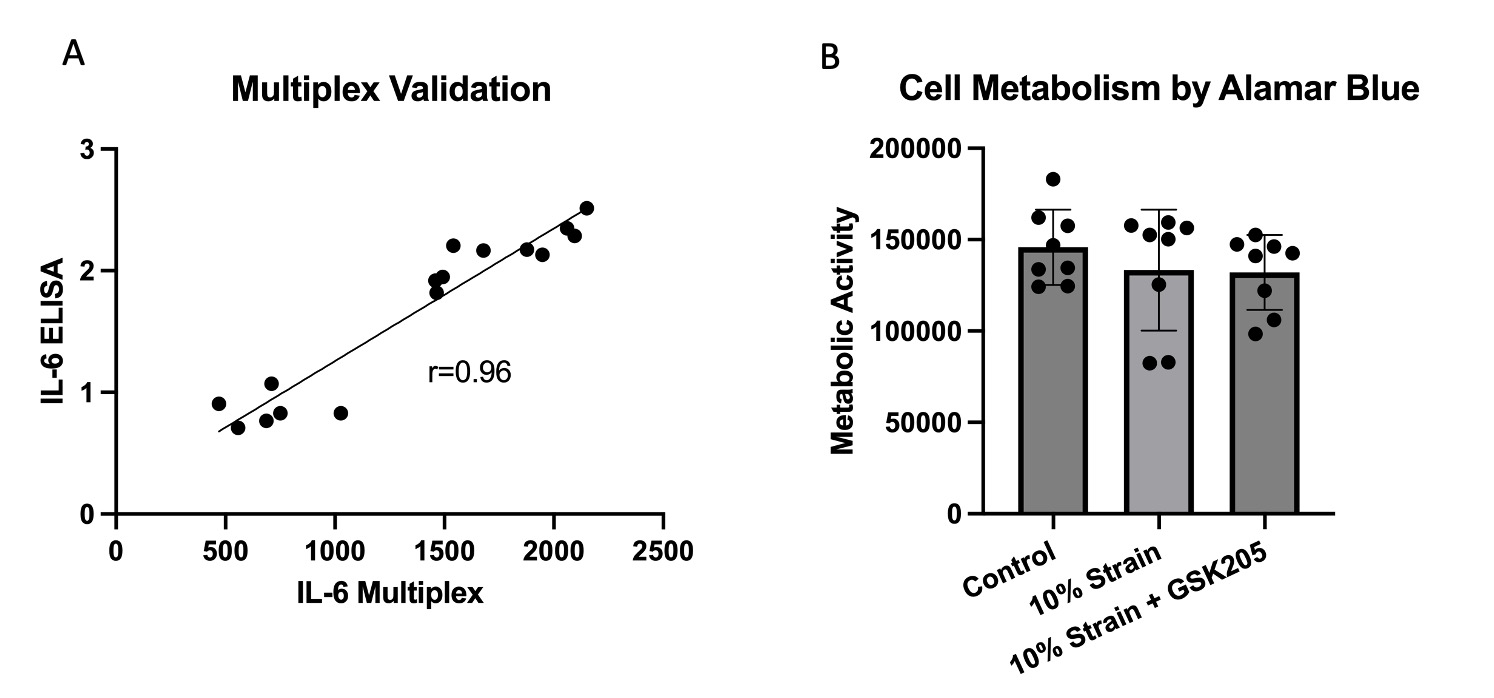
**Supplemental Figure S2:** (A) IL-6 concentration measured by both ELISA and the Luminex-measured are highly correlated (Pearson’s correlation, r=0.96). (B) Dynamic compression did not alter the overall metabolic activity of the IVD organ culture as measure by Alamar Blue assay at Day 7.

**Supplemental Table 1:** Multiplex cytokine panel results for dynamic loading. As dynamic and static samples were sent and analyzed separately, absolute values should not be compared between the two tables.

| \|  \| **Dynamic Average [pg/mL]** \| **Dynamic + GSK205 Average [pg/mL]** \| **p Value** \| \| --- \| --- \| --- \| --- \| \| **Eotaxin** \| 7.11 \| 4.11 \| 0.35 \| \| **G-CSF** \| 1196.92 \| 859.78 \| 0.49 \| \| **GM-CSF** \| 18.61 \| 15.59 \| 0.55 \| \| **IFNγ** \| 0.19 \| low \| N/A \| \| **IL-1α** \| 5.28 \| 1.13 \| 0.03 \| \| **IL-1β** \| 1.40 \| 0.75 \| 0.17 \| \| **IL-2** \| 12.45 \| low \| N/A \| \| **IL-3** \| 0.69 \| 0.66 \| 0.60 \| \| **IL-4** \| 0.07 \| 0.03 \| 0.41 \| \| **IL-5** \| 0.05 \| low \| N/A \| \| **IL-6** \| 6195.04 \| 4358.99 \| 0.10 \| \| **IL-7** \| 0.72 \| 0.45 \| 0.12 \| \| **IL-9** \| 5.66 \| 3.75 \| 0.18 \| \| **IL-10** \| 7.03 \| 0.04 \| 0.22 \| \| **IL-12p40** \| low \| low \| N/A \| \| **IL-12p70** \| 1.44 \| 1.83 \| 0.34 \| \| **IL-13** \| low \| low \| N/A \| \| **IL-15** \| 5.00 \| 4.27 \| 0.58 \| \| **IL-17** \| 0.45 \| 0.06 \| 0.39 \| \| **IP-10** \| 3.67 \| 3.60 \| 0.94 \| \| **KC** \| 5784.83 \| 3745.56 \| 0.10 \| \| **LIF** \| 180.98 \| 150.74 \| 0.33 \| \| **LIX** \| 85.52 \| 23.94 \| 0.04 \| \| **MCP-1** \| 1192.13 \| 675.05 \| 0.11 \| \| **M-CSF** \| 3.97 \| 3.43 \| 0.24 \| \| **MIG** \| 3.22 \| 2.77 \| 0.49 \| \| **MIP-1α** \| 116.83 \| 25.70 \| 0.19 \| \| **MIP-1β** \| 90.87 \| 27.01 \| 0.19 \| \| **MIP-2** \| 2745.42 \| 924.90 \| 0.20 \| \| **RANTES** \| 1.09 \| 0.73 \| 0.46 \| \| **TNFα** \| 1.90 \| 0.63 \| 0.20 \| \| **VEGF** \| 61.53 \| 65.42 \| 0.74 \| \| **6Ckine/Exodus2** \| 150.01 \| 201.11 \| 0.36 \| \| **EPO** \| low \| low \| N/A \| \| **Fractalkine** \| 15.13 \| 20.03 \| 0.37 \| \| **IFNβ-1** \| 16.36 \| 13.47 \| 0.35 \| \| **IL-11** \| 2301.44 \| 2944.75 \| 0.48 \| \| **IL-16** \| 172.11 \| 231.00 \| 0.12 \| \| **IL-20** \| 11.79 \| 10.44 \| 0.62 \| \| **MCP-5** \| 14.09 \| 9.64 \| 0.70 \| \| **MDC** \| 7.39 \| 9.60 \| 0.53 \| \| **MIP-3α** \| 0.93 \| 0.78 \| 0.25 \| \| **MIP-3β** \| 5.63 \| 4.18 \| 0.24 \| \| **TARC** \| 1.26 \| 1.33 \| 0.86 \| \| **TIMP-1** \| 10834.71 \| 14156.82 \| 0.68 \| |
| --- | --- | --- | --- | --- | --- | --- | --- | --- | --- | --- | --- | --- | --- | --- | --- | --- | --- | --- | --- | --- | --- | --- | --- | --- | --- | --- | --- | --- | --- | --- | --- | --- | --- | --- | --- | --- | --- | --- | --- | --- | --- | --- | --- | --- | --- | --- | --- | --- | --- | --- | --- | --- | --- | --- | --- | --- | --- | --- | --- | --- | --- | --- | --- | --- | --- | --- | --- | --- | --- | --- | --- | --- | --- | --- | --- | --- | --- | --- | --- | --- | --- | --- | --- | --- | --- | --- | --- | --- | --- | --- | --- | --- | --- | --- | --- | --- | --- | --- | --- | --- | --- | --- | --- | --- | --- | --- | --- | --- | --- | --- | --- | --- | --- | --- | --- | --- | --- | --- | --- | --- | --- | --- | --- | --- | --- | --- | --- | --- | --- | --- | --- | --- | --- | --- | --- | --- | --- | --- | --- | --- | --- | --- | --- | --- | --- | --- | --- | --- | --- | --- | --- | --- | --- | --- | --- | --- | --- | --- | --- | --- | --- | --- | --- | --- | --- | --- | --- | --- | --- | --- | --- | --- | --- | --- | --- | --- | --- | --- | --- | --- | --- | --- | --- | --- |

**Supplemental Table 2:** Multiplex cytokine panel results for static compression. As dynamic and static samples were sent and analyzed separately, absolute values should not be compared between the two tables.

|  | **Static Average [pg/mL]** | **Static + GSK205 Average**  **[pg/mL]** | **p Value** |
| --- | --- | --- | --- |
| **Eotaxin** | 7.09 | 6.83 | 0.91 |
| **G-CSF** | 6.67 | 7.46 | 0.61 |
| **GM-CSF** | low | 2.24 | N/A |
| **IFNγ** | low | low | N/A |
| **IL-1α** | low | 2.07 | N/A |
| **IL-1β** | 0.18 | 0.41 | 0.04 |
| **IL-2** | low | low | N/A |
| **IL-3** | 0.33 | 0.37 | 0.56 |
| **IL-4** | low | low | N/A |
| **IL-5** | low | low | N/A |
| **IL-6** | 155.07 | 132.34 | 0.63 |
| **IL-7** | 0.91 | 0.55 | 0.08 |
| **IL-9** | 2.47 | 2.22 | 0.85 |
| **IL-10** | low | low | N/A |
| **IL-12p40** | low | low | N/A |
| **IL-12p70** | 1.03 | 1.47 | 0.31 |
| **IL-13** | low | low | N/A |
| **IL-15** | 6.41 | 5.73 | 0.71 |
| **IL-17** | low | low | N/A |
| **IP-10** | 7.56 | 5.31 | 0.28 |
| **KC** | 619.73 | 566.81 | 0.67 |
| **LIF** | 9.37 | 6.41 | 0.24 |
| **LIX** | low | low | N/A |
| **MCP-1** | 788.82 | 819.06 | 0.87 |
| **M-CSF** | 1.86 | 2.01 | 0.75 |
| **MIG** | 5.03 | 4.17 | 0.39 |
| **MIP-1α** | 6.35 | 8.98 | 0.57 |
| **MIP-1β** | 3.51 | 6.32 | 0.14 |
| **MIP-2** | 21.68 | 26.47 | 0.19 |
| **RANTES** | 1.19 | 1.25 | 0.82 |
| **TNFα** | low | low | N/A |
| **VEGF** | 322.75 | 204.86 | 0.15 |
| **6Ckine/Exodus2** | 132.90 | 138.76 | 0.82 |
| **EPO** | low | low | N/A |
| **Fractalkine** | 29.78 | 24.69 | 0.58 |
| **IFNβ-1** | 19.16 | 19.64 | 0.82 |
| **IL-11** | 217.15 | 160.44 | 0.41 |
| **IL-16** | 10.43 | 8.32 | 0.47 |
| **IL-20** | 14.87 | 12.65 | 0.37 |
| **MCP-5** | 5.38 | 6.09 | 0.83 |
| **MDC** | 0.85 | 0.63 | 0.28 |
| **MIP-3α** | 0.91 | 0.93 | 0.85 |
| **MIP-3β** | 6.83 | 5.36 | 0.19 |
| **TARC** | 1.80 | 1.29 | 0.28 |
| **TIMP-1** | 9232.89 | 46002.89 | 0.33 |

**Supplemental Table 3**: Control correlations
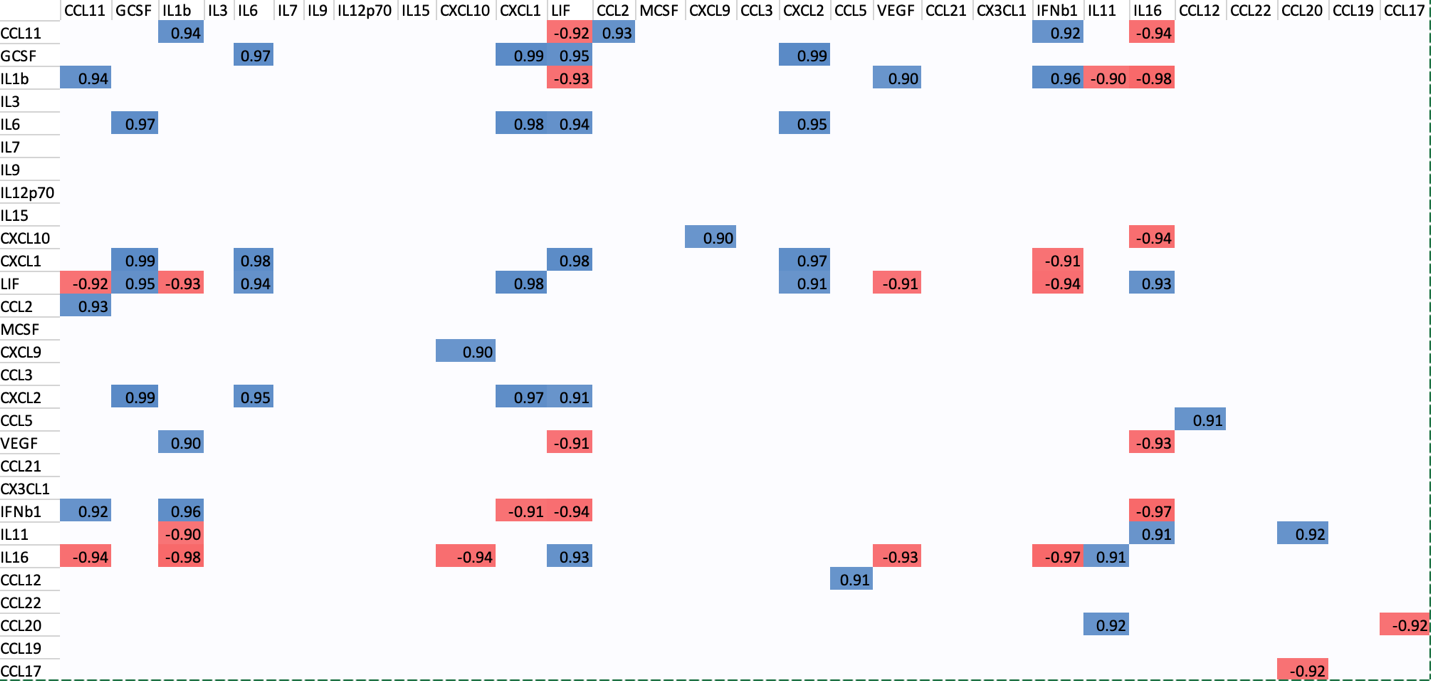


**Supplemental Table 4**: Static Compression correlations


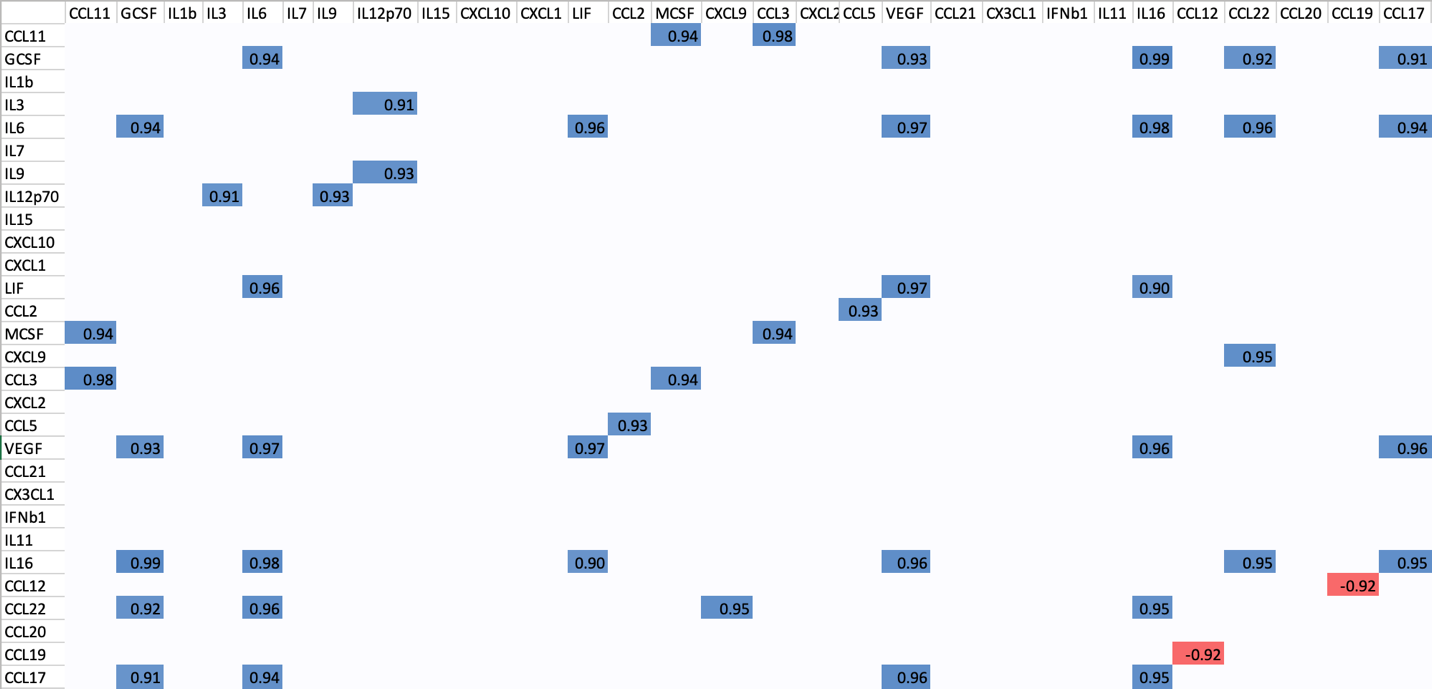


**Supplemental Table 5**: Static Compression + GSK205 correlations


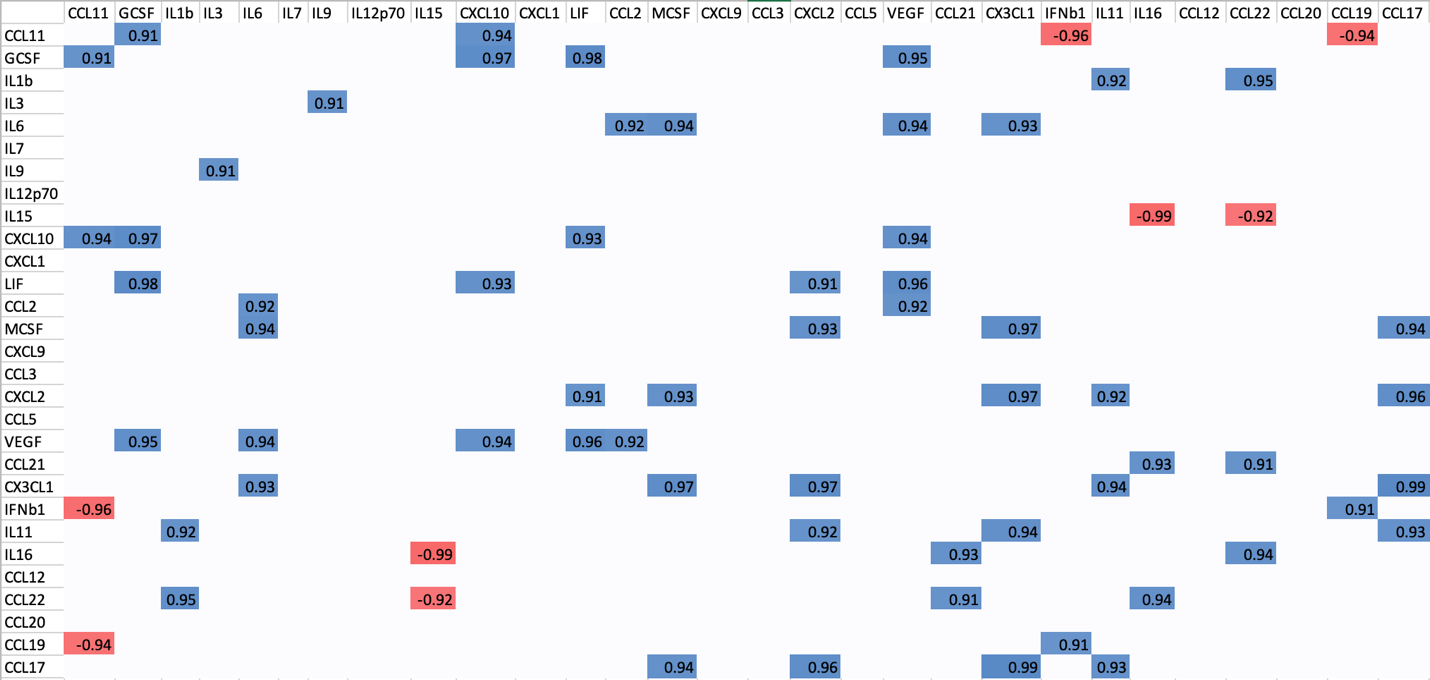


**Supplemental Table 6**: Dynamic Compression correlations


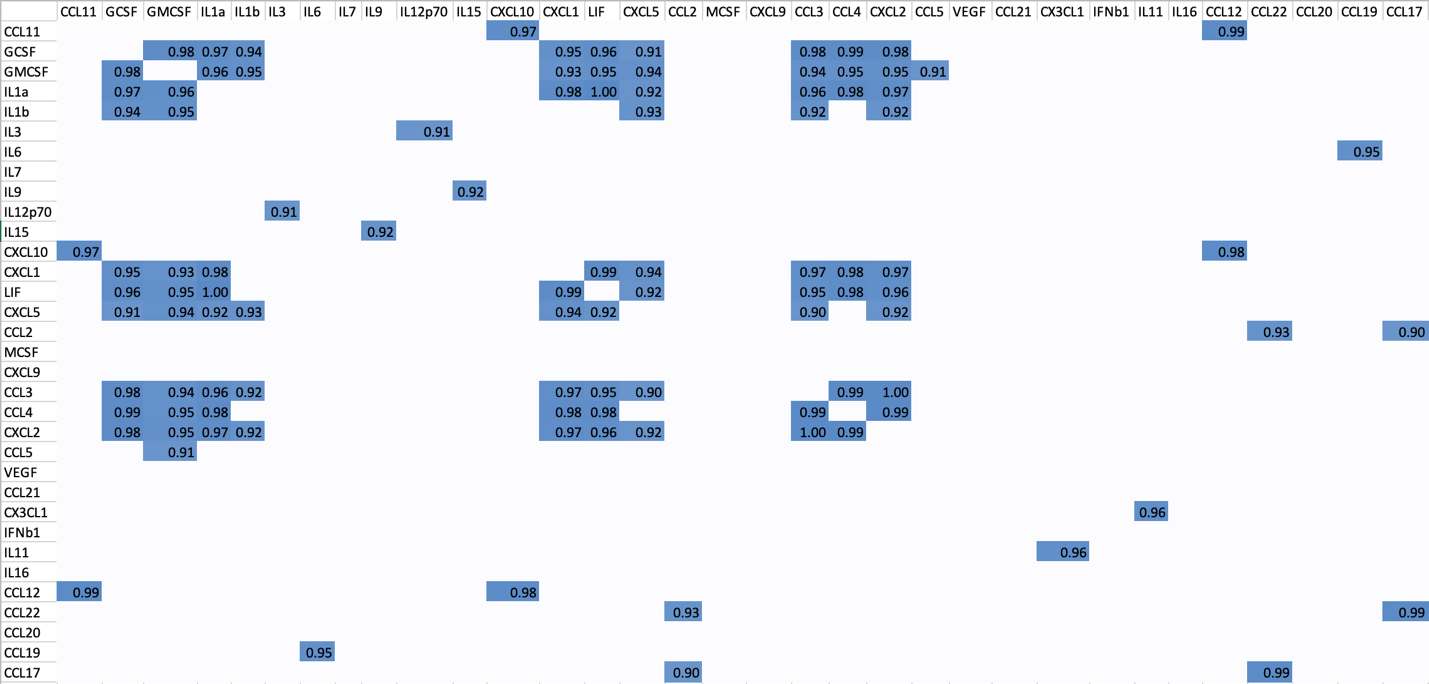


**Supplemental Table 7**: Dynamic Compression + GSK205 correlations


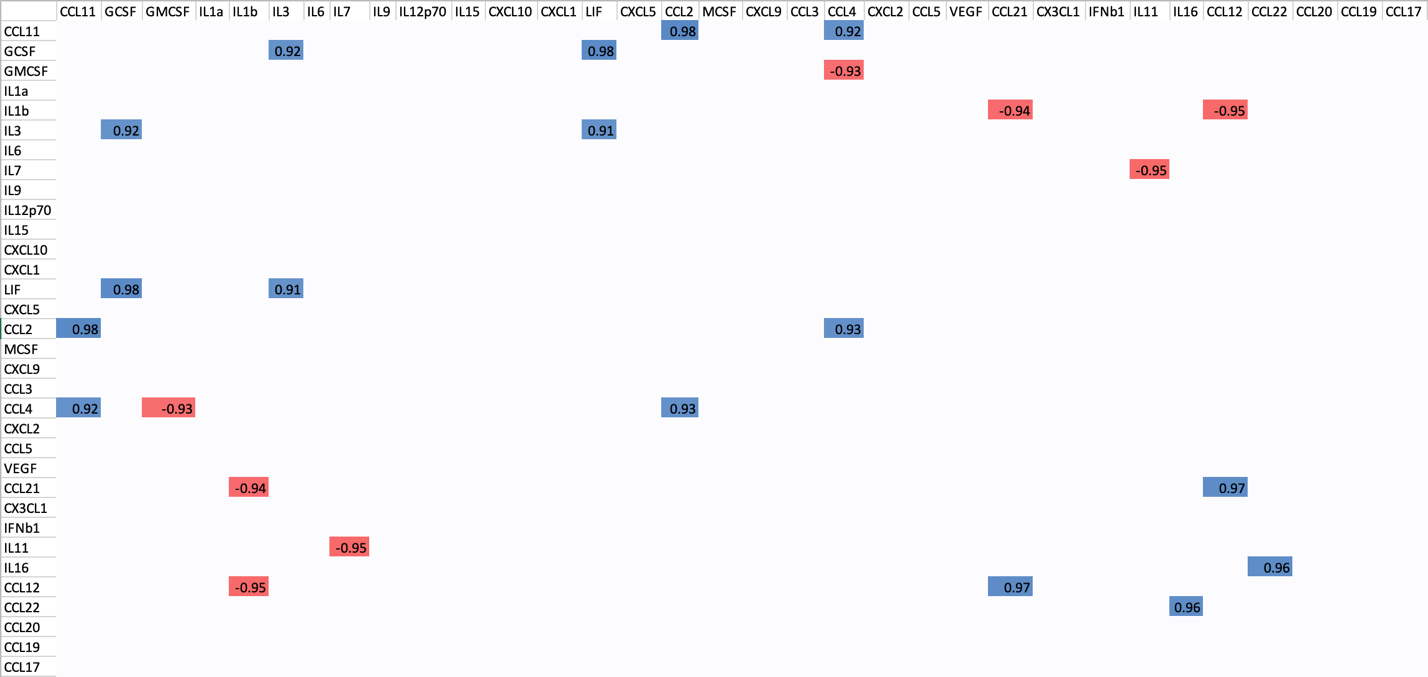


**Supplemental Table 8**: TRPV4 activation – Day 3


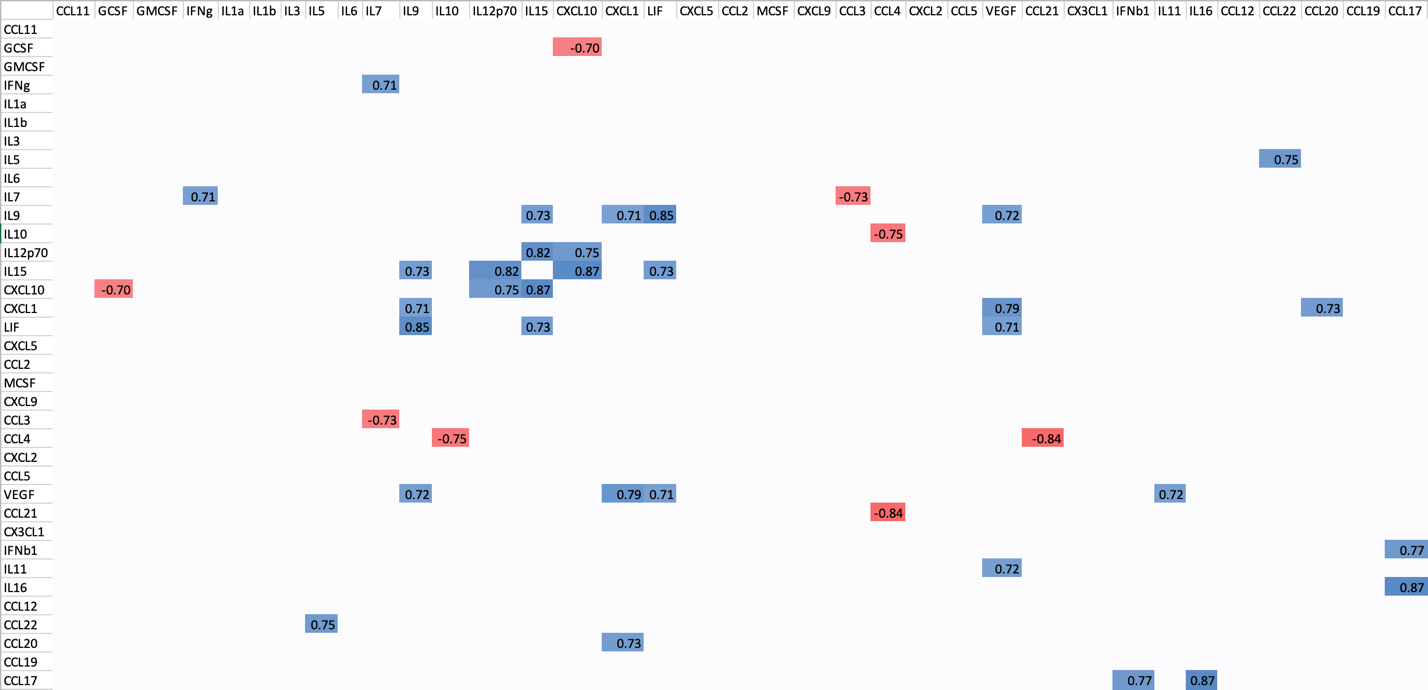


**Supplemental Table 9**: TRPV4 activation – Day 7


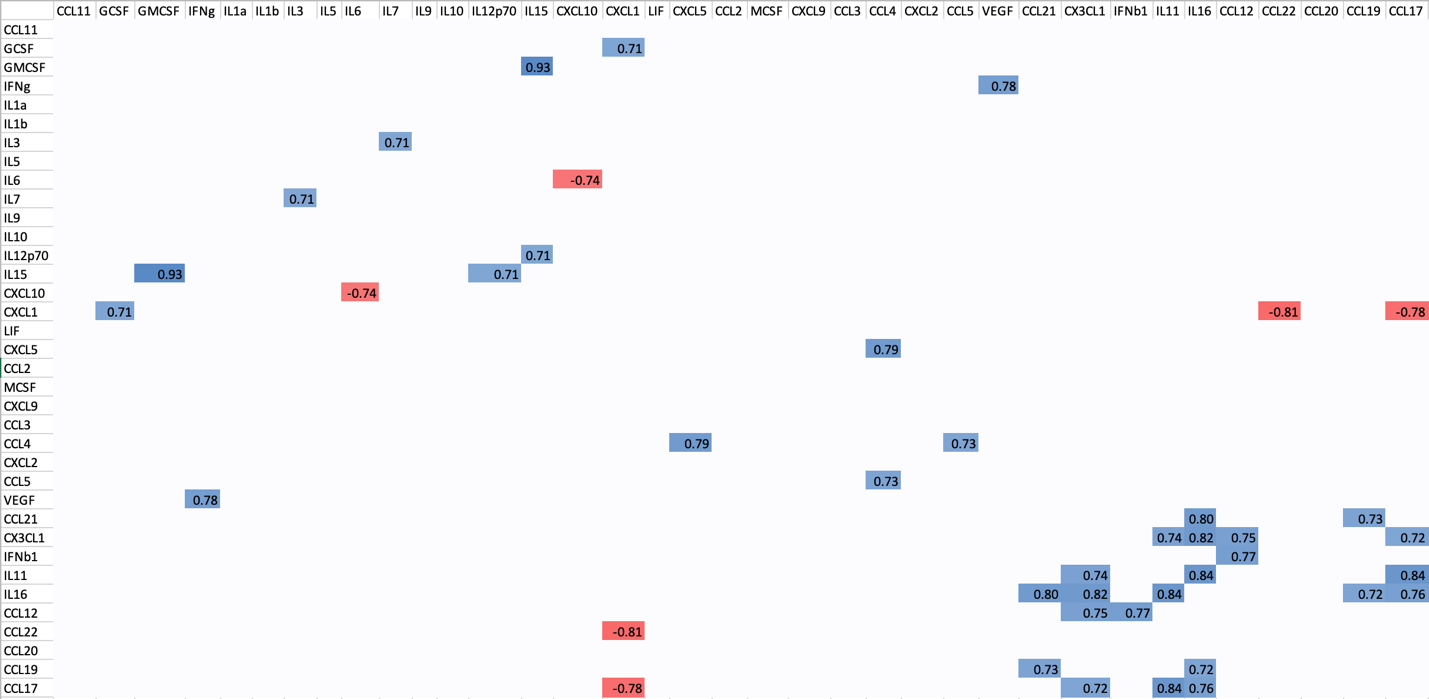

Supplement: Supplementary file 1 — DATA S1. Supporting Information. [file JSP2-6-e1282-s001.docx]
